# POPULATION AGING AND INCREASING CHRONIC DISEASE BURDEN AMONG UNAUTHORIZED AGRICULTURAL WORKERS

**DOI:** 10.1093/geroni/igae098.3880

**Published:** 2024-12-31

**Authors:** Nicholas Bishop, Baltazar Campos, Daniel Martinez, Jenjira Yahirun

**Affiliations:** University of Arizona, Tucson, Arizona, United States; University of Arizona, Tucson, Arizona, United States; University of Arizona, Tucson, Arizona, United States; Bowling Green State University, Bowling Green, Ohio, United States

## Abstract

The US and Mexico are experiencing rapid population aging and increased chronic disease burden. We hypothesize these dynamics are magnified among undocumented farmworkers who work in challenging conditions and have limited access to healthcare. We used data from the National Agricultural Workers Survey (2001–2018) to examine whether 1) the age of undocumented farmworkers has increased over time (n=19,434), and 2) chronic disease burden has increased among undocumented workers aged 50+ (n=1,463). Multiple regression was used to examine the association between respondent age and survey year, and Poisson regression was used to estimate the association between number of doctor-diagnosed conditions (asthma, diabetes, hypertension, and heart disease) and survey year among respondents aged 50+. Models were adjusted for covariates (sex, migrant type, health service use, region, and age (in the chronic disease model)), and person weights. The mean age of the sample in survey years 2001–2002 was 28 (SD=9.7), which increased to 39 (SD=11.0) by 2017–2018. After adjusting for covariates and weights, there was a monotonic positive increase in age across survey years (e.g., respondents surveyed in 2017–2018 were on average 8.36 years older (95% CI:7.61–9.12, p<.001) than those surveyed in 2001–2002). Among undocumented agricultural workers aged 50+, respondents in the 2017–2018 survey waves had an expected condition count 202% greater than respondents in the 2001–2002 waves (IRR=3.02, 95% CI:1.45–6.29, p=0.003). An aging population of undocumented farmworkers with increasing chronic disease burden represents a significant health equity issue and strain on US agricultural output.

